# Future of nature, our future. A preregistered report on future time perspective, social value orientation, and pro-environmental outcomes based on data from Poland and Sweden

**DOI:** 10.3389/fpsyg.2023.1217139

**Published:** 2023-12-01

**Authors:** Iwona Nowakowska, Michael Rönnlund

**Affiliations:** ^1^Institute of Psychology, Maria Grzegorzewska University, Warsaw, Poland; ^2^Department of Psychology, Umeå University, Umeå, Sweden

**Keywords:** future time perspective, moral foundations, pandemic threat, pro-environmental behaviors, social value orientation

## Abstract

**Introduction:**

The objective of the study was to examine the role of social value orientation and future time perspective to account for individual differences in pro-environmental behaviors, intentions, and opinions about the link between pro-environmental action and pandemic threat (three separate models) in Polish and Swedish samples expected to differ in rate of pro-environmental behaviors (higher in Sweden). We hypothesized that for Poland, future time perspective would be linked to pro-environmental outcomes only when social value orientation is average or high. In contrast, for Sweden, we expected a significant link between these variables regardless of social value orientation.

**Methods:**

In total, 301 (150 Polish, 151 Swedish) participants completed online surveys via Prolific.co research panel. We controlled for individualizing/binding moral foundations, present time perspectives, and selected demographic variables in the analyses.

**Results:**

In line with expectations, the individualizing moral foundations were a significant predictor across all three models. The data did not support our focal hypothesis regarding the interaction between future time perspective and social value orientation. For pro-environmental behaviors in the past 6 months, the future time perspective was a predictor only when social value orientation was low.

**Discussion:**

The results suggest that when encouraging more competitive (compared to altruistic) people to behave in a green way, it might be crucial to underline the future consequences and benefits, consistent with the future time perspective. The pro-environmental campaigns could, therefore, highlight how green behavior may bring personal gains in the future, which are typically valued by individualistic people, such as savings or social status.

## Introduction

The world faces a climate change crisis (Romanello et al., 2022). Without taking action, humanity is threatened with many adverse effects, such as diseases (Khraishah et al., 2022; Semenza et al., 2022), crop failure (Kogo et al., 2022), extreme temperatures, and hazardous weather conditions (Clarke et al., 2022). Furthermore, due to the emergence of the COVID-19 pandemic, research was conducted regarding the relationships between the environmental state and the risk of spreading viruses. A study from Germany indicated that not only temperature but also the presence of PM2.5, O_3_, and NO_2_ was associated with the spread of COVID-19, whereas PM10, humidity, and environmental quality index were significantly related to the number of active COVID-19 cases (Bilal et al., 2020). Another study from California showed that PM2.5, PM10, NO_2_, CO, and SO_2_ were significantly associated with COVID-19 cases (Bashir et al., 2020). It provides preliminary data on how pollutants bring a risk of spreading viruses and how they might contribute to pandemics.

Success in combatting climate change relies on governmental and individual actions (Maiella et al., 2020). Therefore, exploring the individual characteristics that promote pro-environmental behaviors, intentions, and beliefs regarding the link between pro-environmental attitudes and pandemics' emergence is crucial. Without effort and consideration of the future consequences of the present actions by individual persons (Ho et al., 2020), any large-scale pro-environmental policy cannot be successfully implemented.

Based on the social norm activation model by Schwartz (1970) and later literature taking temporal and social aspects in prosocial behaviors into account (Joireman et al., 2001), the purpose of the current study is to identify individual difference variable responsible for considering the long-term consequences of actions (future time perspective) and the welfare of other people (social value orientation). We also aim to test whether their interaction accounts for (1) pro-environmental behaviors during the last 6 months, (2) pro-environmental behavior intentions in the following 6 months, and (3) opinion about the linkage between pro-environmental attitude and the threat of pandemics.

Given the difference in Polish and Swedish policies and the pro-environmental culture (Mikuła et al., 2021), we chose these two countries to additionally check if the participant's country of residence modifies this interaction mechanism. Although future time perspective is regarded as a trait-like and relatively stable factor (Kairys and Liniauskaite, 2015), how it links to behaviors may differ depending on what is valued in a particular culture as having long-term positive consequences. For instance, pro-environmental behavior might be seen as a waste of money when it is deemed costly, and the public needs to be convinced about its long-term benefits or investment when its positive results are precise. A future-oriented person might choose what they find beneficial, and the appraisal of behavior as such depends on general beliefs in their social circle (e.g., in their own country).

The inclusion of moral foundations and present time perspectives as covariates will enable us to determine whether the future time perspective effect is noticeable over more general moral attitudes of caring for other people and caring for the present over the future. We will also test the potential effects of sociodemographic variables: age, gender, socioeconomic status, and education.

By our study, we aim to address theoretical gaps regarding (1) the intercultural differences between countries of low (Poland) and high (Sweden) pro-environmental behaviors in society; (2) testing the norm activation model (Schwartz, 1970) in the pro-environmental context, taking into account present time perspectives (which complement the understanding of future time perspective and have been recently proven to be essential for prosocial outcomes; Maki et al., 2016; Nowakowska, 2023, including pro-environmental ones, Wittmann and Sircova, 2018), moral foundations (which describe not only caring for the welfare of others does social value orientation but also the moral reasons for behaviors and the underpinnings of politics-related convictions; Graham et al., 2009), and sociodemographic covariates.

Our analysis is expected to broaden knowledge about the norm activation model and test whether time perspectives, recently gaining attention in the field of pro-environmental behaviors (Hoffmann et al., 2022; Olsen et al., 2023), remain significant predictors of such activities, when controlling for the propensity to care for others and morality. Our results might also prove vital for pro-environmental education and campaigns, for instance, how to shape attitudes and behaviors—what aspects we should target (e.g., future orientation, cooperation tendencies, or morality). To our knowledge, no such intercultural comparison has been made regarding pro-environmental behaviors and intentions based on our theoretical basis and this set of covariates.

### Literature review

People are often supposed to choose between long-term and short-term interests—for themselves, society, or interaction partners (Milfont and Gouveia, 2006). The norm activation model (Schwartz, 1970) states that personal norms (with altruistic values highlighted) influence behaviors only when a person thinks that their action has consequences for another's wellbeing (as other people are the main objects valued by altruists) and when an individual believes in their responsibility in doing these actions. Personal norms are forming a feeling of moral obligation to either undertake or refrain from particular actions (Schwartz and Howard, 1981). Stern et al. (1993) and Stern (2000), in his value-belief norm model, suggested an expansion of Schwartz's model. Value orientation, according to this conceptualization, describes the principle that guides the desirable states or outcomes and is hypothesized to influence the way how people formulate and structure their beliefs regarding the environment (Stern, 2000).

Pro-environmental behaviors are considered one of the most important in terms of their consequences for society. Utilizing resources as much as possible can bring short-term benefits for an individual but harm society, whereas sparing these resources can bring long-term benefits to society. Joireman et al. (2001) present pro-environmental behavior through the norm activation model as a social dilemma embedded in two dimensions—the social dilemma (conflict between interests of the self and others) and the temporal one (immediate and delayed consequences of action). Based on available data, we propose that social value orientation in solving the social dimension of the social dilemma of undertaking pro-environmental behavior and the future time perspective can be critical in solving the temporal dimension (Joireman et al., 2001; Maki et al., 2016). Moreover, we suggest that it is important to control for moral foundations, as pro-environmental behaviors can be linked to moral norms and resulting political orientations (Chan and Bishop, 2013; Vainio and Mäkiniemi, 2016; Milfont et al., 2019).

### Pro-environmental behaviors in Poland and Sweden

Countries, even those close geographically, differ significantly in the level of pro-environmental behaviors the citizens display. It is due to historical, socioeconomic, and mentality-related aspects. Poland and Sweden are interesting examples of such a difference. Poland is a country with <20,000 USD gross domestic product per capita. It has <10% usage of renewable energy sources (Iwińska et al., 2023). Poland is also still developing regarding environmental protection and resists the shift from conventional energy sources (Zuk, 2022). The country was the only one to reject the Green Deal, which aimed to introduce the rules of a climate-neutral economy by 2050. In 2017, decisions were made regarding logging the Bialowieza Forest—a unique natural treasure of the Polish territory—resulting in worldwide protests (Cislak et al., 2021). It is also one of Europe's countries with the lowest rates of pro-environmental behaviors (Mikuła et al., 2021); however, during the COVID-19 pandemic, it was ranked as showing higher rates of pro-environmental behaviors than Sweden (Iwińska et al., 2023). By contrast, Sweden is a relatively rich European country with over 50,000 USD in gross domestic product per capita. It has over 40% usage of renewable energy sources (Iwińska et al., 2023). Sweden is at the top of the green policies in Europe (D'Adamo et al., 2020). It showed the highest rates of pro-environmental behaviors in Europe (Mikuła et al., 2021), but during COVID-19—had the rate of pro-environmental behaviors lower than Poland (Iwińska et al., 2023). Nevertheless, Sweden is considered a leader in green regulations and actions (Berck et al., 2011).

According to a study by Mikusiński et al. (2023), value orientations are one of the most important factors associated with human-nature connectedness (related to pro-environmental attitudes; Klaniecki et al., 2018). However, a study by De Groot and Steg (2007) showed that value orientations were strongly related to personal norms and awareness of consequences only in the case of Sweden (and not in the case of four other investigated countries: the Netherlands, Italy, Austria, and the Czech Republic). On the contrary, in a study by Caniëls et al. (2021), altruistic value orientation was unrelated to pro-environmental behaviors in the case of Poland. This suggests that both countries may also differ in the case of the role of individual differences in pro-environmental behaviors.

### The social dilemma of pro-environmental behaviors: social value orientation

The individual endorsement of social norms describing the preferred consequences of one's own actions may be operationalized with social value orientation (Joireman et al., 2001). Social value orientation is a personal trait (Messick and McClintock, 1968), which describes the preference for self or other's outcomes in social interactions (Van Lange, 2000). It may be considered a continuum regarding the individual's tendency for rivalry or altruism in sharing resources with others (Murphy et al., 2011). Generally, higher social value orientation, i.e., altruism, facilitates cooperation in social dilemmas (a meta-analysis by Pletzer et al., 2018). A pro-environmental behavior is a social dilemma (Bogaert et al., 2008). It has been proven that the higher the altruistic value orientation, the higher the pro-environmental self-determination (De Groot and Steg, 2010). Social value orientation has been used as an operationalization of the personal norms (altruistic concerns) within the norm activation model framework (Joireman et al., 2001).

Based on the literature review above, first, we hypothesized that:

(H1) Social value orientation is positively related to pro-environmental behaviors, intentions, and opinions about the pro-environmental behaviors-pandemic risk linkage (the latter referred to below as “pro-environmental opinions”).

### The temporal dilemma of pro-environmental behaviors: future time perspective

Time perspective is a dimension of the psychological time construal in humans. It is a result of cognitive processes which divide personal experiences into past, present and future temporal frames (Zimbardo and Boyd, 1999). Time perspectives describe to what extent people take into account their past, present, or future when making decisions (Zimbardo and Boyd, 2008). One of the traditionally distinguished time perspectives—the future time perspective -involves planning and being able to consider consequences that overcome the immediacy and the present (Zimbardo and Boyd, 1999). However, this perspective typically involves caring for oneself and one's own future, not the collective one (Zimbardo and Boyd, 2008). Despite this, future time perspective has been positively and consistently linked to pro-environmental behaviors, and the link was stronger for behaviors than attitudes (see meta-analysis by Milfont et al., 2012). Sustainable behaviors require anticipation of consequences and long-term orientation. Therefore, the relationship between future time perspective and sustainable behaviors exists, as future-oriented people are good at planning and meeting obligations in the long term (Corral-Verdugo and Pinheiro, 2006). Future-oriented people also have the ability to visualize their objectives, which has an impact on their present decisions (Keough et al., 1999), which supports them in undertaking pro-environmental behaviors (Corral-Verdugo et al., 2006).

Future time perspective can also facilitate solving the temporal dimension of the social dilemma in a way that promotes positive consequences for oneself or society (Arnocky et al., 2014). The motivation to act pro-environmental stems from the extent to which the consequences of pro-environmental action match the things people value (Stern et al., 1993). Based on the abovementioned data, the future time perspective, due to its relation to considering future consequences of actions, might be viewed as boosting the ability for consequence awareness, as conceptualized by Schwartz (1970). In our study, we propose that the effect of future time perspective on pro-environmental behaviors/intentions/opinions depends on what consequences (benefits for oneself or others) the individual values.

We suppose that in the case of Poland, where the pro-environmental culture is not embedded, the future time perspective requires social value orientation to display behaviors that care for the collective (e.g., the pro-environmental ones). In the case of Sweden, where the green culture is more embedded in everyday life (as this country ranks very high in sustainability indices, e.g., Sustainable Development Report, 2021), we hypothesize that the simple effect of future time perspective and social value orientation will be present. However, future time perspective will be linked to pro-environmental behaviors, intentions, and opinions about the pandemic threat stemming from a lack of pro-environmental behaviors regardless of the level of social value orientation (thus, the interactive effect will not be found). In sum, we state two further hypotheses:

(H2) In the Polish sample, the future time perspective requires at least an average social value orientation to be related to pro-environmental behaviors/intentions/opinions.
(H3) In the Swedish sample, future time perspective is linked to pro-environmental behaviors/intentions/opinions regardless of the level of social value orientation (thus, the interactive effect will not be found).

### Moral foundations as covariates of the social and temporal dilemma

As the norm activation model implies, moral norms may influence behaviors (Schwartz, 1970). Therefore, we plan to involve moral foundations as variables that should be controlled in the regression model. Moral foundations are constructs that are intuitively and unconsciously activated in any situation encountered by a person (Graham et al., 2011; Dickinson et al., 2016) and have an impact on judgment and behavior (Haidt and Graham, 2007). Moral foundations enable us to explore the behavioral orientation toward sharing with others (as does social value orientation, Van Lange et al., 2007; Murphy et al., 2011, but from an angle of moral appraisal, not a preference for own or other's outcomes in social interactions) and the underlying moral motivation of behavior, as described by Schwartz's theory (Schwartz, 1970). Moral foundations have been proven vital in predicting pro-environmental outcomes (Skalski-Bednarz et al., 2023). Moral foundations are classified into individualizing (harm avoidance and fairness/reciprocity) and binding (caring for ingroup loyalty, respect for authorities, and purity and sanctity; Graham et al., 2009). Typically, individualizing morality is considered the one that predicts a liberal orientation, and binding—predicts a conservative orientation (Van Leeuwen and Park, 2009). Individualizing morality and liberal attitudes promote pro-environmental attitudes (Vainio and Mäkiniemi, 2016; Milfont et al., 2019), whereas the binding morality and conservative worldview are linked to decreased level of pro-environmental choices (Vainio and Mäkiniemi, 2016). We suppose that:

(H4) Individualizing values may be positive. In contrast, the binding values are negatively linked to behaviors, intentions, and consideration of a pandemic as a threat reliant on the environment.

### Present time perspectives and pro-environmental outcomes

We will also enter present time perspectives as covariates to our model to control for the effect of the endorsement of the present in pro-environmental behaviors (Arnocky et al., 2014; Valizadeh et al., 2018). Present-hedonistic time perspective relates to a preference for joy and seeking pleasure in present behavior (Zimbardo and Boyd, 1999). It is negatively related to pro-environmental behavior due to the impulsivity associated with it (Wittmann and Sircova, 2018). Impulsivity limits the capacity to plan and act for the sake of long-term consequences, and that is presumably why pro-environmental behaviors may be challenging for people with high levels of this trait. Moreover, at present, hedonism is linked to seeking pleasure, and sustainable behaviors might be linked to the devotion of own pleasure for positive results for the general public. The present-fatalistic time perspective may also encourage the use of natural resources here and now without concern for the future (Corral-Verdugo et al., 2006). Present fatalism is related to a conviction that an individual does not have the force to influence the course of life (Zimbardo and Boyd, 1999), which may lead to a decreased sense of responsibility for own behaviors and the environment.

### Demographic variables and pro-environmental outcomes

Relevant demographic variables are as follows: age, gender, education, and socioeconomic status will also be controlled. In literature, minor effects of older age on sustainable behaviors are found (see meta-analysis by Wiernik et al., 2013). Studies have also shown women to be more likely to engage in pro-environmental behaviors. Higher socioeconomic status (measured as education and income level) facilitates such behaviors (Chen et al., 2011; Otto et al., 2016; Patel et al., 2017; Casaló and Escario, 2018). Education has also been described as increasing the propensity for environmentally friendly behavior, presumably due to higher concern for social welfare in educated people (Meyer, 2015).

### Graphical summary of the hypothesized paths

**Figure 1:**
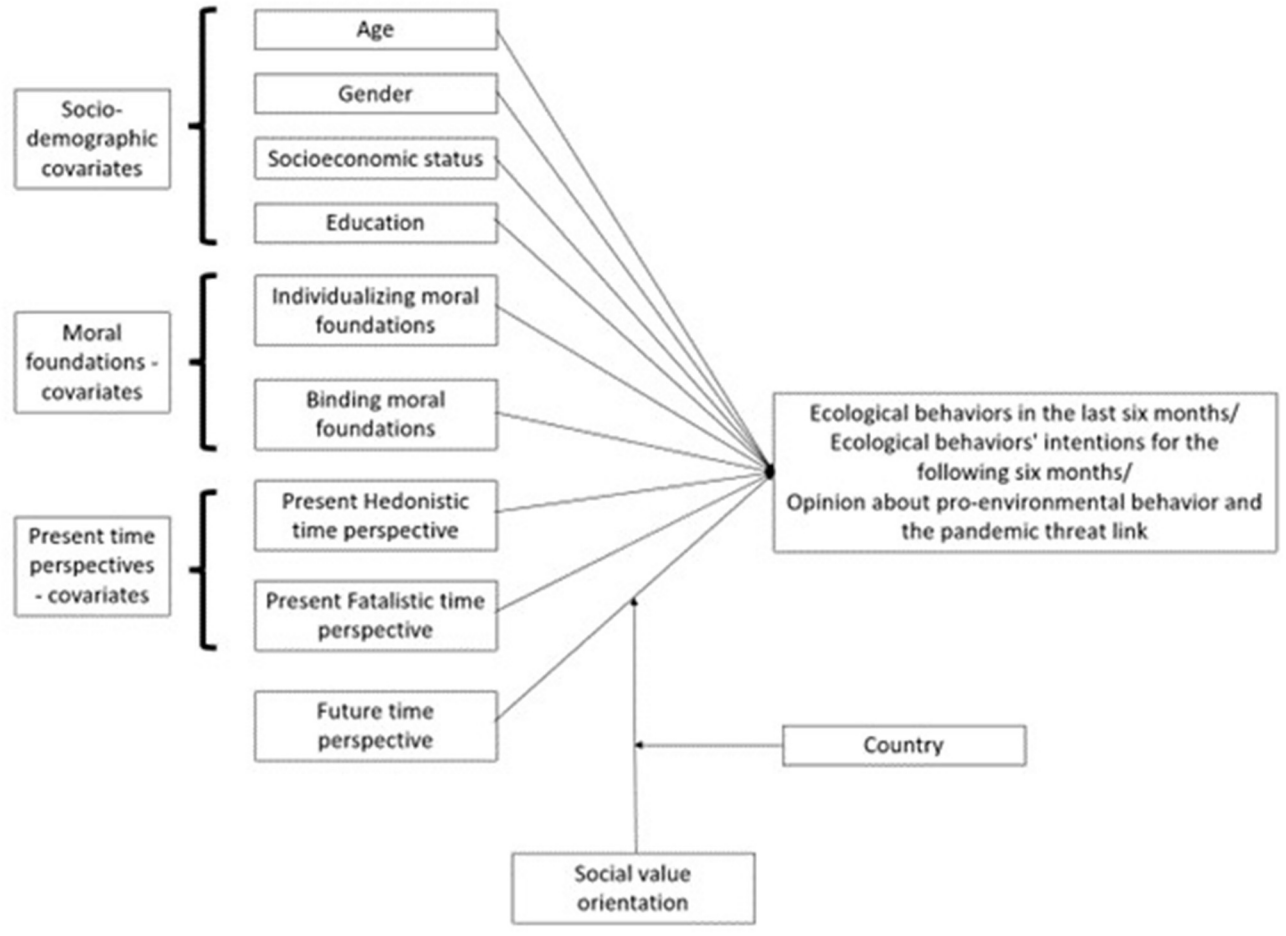
Visualization of the hypothesized paths to be tested in three models.

Figure 1 shows a visualization of the hypothesized paths to be tested in three models predicting: ecological behaviors in the last 6 months, ecological behaviors' intentions for the following 6 months, and opinions about pro-environmental behavior and the pandemic threat risk.

## Materials and methods

### Power analysis and preregistration-related demands

As indicated in our preregistration, we aimed to recruit *N* = 150 participants from Poland and *N* = 150 from Sweden (total *N* = 300). A power analysis conducted in G^*^Power 3.1.9.4 (Faul et al., 2009) suggested that such sample size will allow us to detect an effect of 0.10 with defined α = 0.05 with a power of 0.95 in a regression analysis with 15 predictors. Our goal was to recruit participants from an age range of 18–65.

### Participants

#### Polish sample

In total, 150 participants aged 18–57 (*M* = 25.45; SD = 7.55) participated in the study (74 women, 49.3%, 73 men, 48.7%, three of other gender/refusing to answer, 2.0%). A total of 86 (57.3%) were currently employed, and 64 (42.7%) were not. Concerning relationship status, 15 (10.0%) were married, 65 (43.3%) were in an informalized relationship, 64 (42.7%) were single, and 6 (4.0%) declared another status. For the place of residence, 24 (16.0%) lived in a village, 21 (14.0%) in a town with up to 50,000 inhabitants, 17 (11.3%) in a town with 50,000–100,000 inhabitants, 40 (26.7%) in a town with 100,000–500,000 inhabitants, and 48 (32.0%) in a town with 500,000 inhabitants or more. On a scale from 0 to 10, where 0 meant *I cannot afford basic expenses* and 10 = *I can afford whatever I want and can save money*, the answers ranged from 1 to 10 (*M* = 5.81; SD = 1.74). For education, the range of education (years spent on actual learning, parallel studying not included) was 11–21 (*M* = 14.99; SD = 2.13). A total of 133 participants were meat consumers (88.7%).

#### Swedish sample

A total of 151 participants aged 18–63 (*M* = 31.32; *SD* = 9.81) participated in the study (54 women, 35.8%, 96 men, 63.6%, 1 of other gender/refusing to answer, 0.7%). A total of 106 (70.2%) were employed, and 45 (29.8%) were not. For the relationship status, 27 (17.9%) were married, 42 (27.8%) were in an informalized relationship, 77 (51.0%) were single, and five (3.3%) declared another status. For the place of residence, 31 (20.5%) lived in a village, 22 (14.6%) in a town with up to 50,000 inhabitants, 31 (20.5%) in a town with 50,000–100,000 inhabitants, 36 (23.8%) in a town with 100,000–500,000 inhabitants, and 31 (20.5%) in a town with 500,000 inhabitants or more. On a scale from 0 to 10, where 0 meant *I cannot afford basic expenses* and 10 *I can afford whatever I want and can save money*, the answers ranged from 0 to 10 (*M* = 5.52; *SD* = 2.36). For education, the range of education (years spent on actual learning, parallel studying not included) was 5–26 (*M* = 14.84; *SD* = 2.99). A total of 122 (80.8%) participants were meat consumers.

### Procedure

The study was performed online and was fully questionnaire-based. All questions were multiple choice (no open-ended questions).

We used Qualtrics (Qualtrics, Provo, UT) for survey design. All data were collected using prolific.co (previously prolific.ac), a subject pool for online studies. Prolific is a reliable source of participants (Palan and Schitter, 2018). Its advantage is the availability to recruit people of different nationalities using the same rules of study inclusion and remuneration. All participants who finished the survey were remunerated through Prolific with a small financial reward (equal for all). As the study relied on convenience sampling, the sociodemographic structure was not representative of the whole population.

We started the data collection in Poland and Sweden simultaneously (end of June 2022). For Poland, the data collection ran till the end of June 2022; for Sweden, it finished in October 2022. We aimed to recruit the number of participants specified in our preregistration. The difference in the length of data collection was due to the smaller number of registered participants from Sweden than from Poland.

### Measures

We used the same instruments for the Polish and Swedish samples. Except for the part on ecology and pandemic threat, which were prepared on our own, the measures were initially published in English and validated in Polish and Swedish.

#### Zimbardo Time Perspective Inventory

Zimbardo Time Perspective Inventory was used to measure time perspectives (original version: Zimbardo and Boyd, 1999; Polish version: Przepiórka, 2011; Swedish version: Carelli et al., 2011). It is a self-report measure consisting, in the Polish version, of five subscales: future, present-hedonistic, present-fatalistic, past-positive, and past-negative (56 items), and the Swedish version, six subscales: same as in the Polish version plus future-negative (64 items). For the current study, only future (e.g., *When I want to achieve something, I set goals and consider specific means for reaching those goals*), Present-Hedonistic (e.g., *I believe that getting together with one's friends to party is one of life's important pleasure*), and Present-Fatalistic (e.g., *Fate determines much in my life*) were the subscales of interest. The participants answered on a 5-point Likert scale, ranging from *very uncharacteristic of me* (coded as 1) to *very characteristic of me* (coded as 5). The general scores for the subscales of interest were computed as the mean of relevant items. For the Polish sample: for the future time perspective, Cronbach's α = 0.82; for present-hedonistic time perspective α = 0.79; for present-fatalistic time perspective α = 0.69. For the Swedish sample: for future time perspective, Cronbach's α = 0.72; for present-hedonistic time perspective, α = 0.83; for present-fatalistic time perspective, α = 0.72.

#### Social Value Orientation Slider Measure

Social Value Orientation Slider Measure was used to assess social value orientation (original version: Murphy et al., 2011; Polish and Swedish versions taken from the international project materials by Froehlich et al., 2021). Six basic items assessing the continuum of social value orientation were used. The participants are asked to imagine they need to allocate resources through money payoff between themselves and a stranger. The measure is a decomposed game; each item reflects specific payoff allocations. The results are called the social value orientation angle and can be computed using a syntax by Baumgartner (*n*/*d*). The angle can take values from −16.26 (extremely competitive) to 61.39 (extremely altruistic). The measure enables classifying each participant into a category of competitive, individualistic, prosocial, or altruistic player (based on angle cutoff points) and obtaining a continuous score for relevant computations. In the current samples, among Poles, none of the participants was competitive, 33 (22.0%) individualistic, 116 (77.3%) prosocial, 1 (0.7%) altruistic; among Swedes, 2 (1.3%) participants were competitive, 21 (13.9%) individualistic, 126 (83.4%) prosocial, 2 (1.3%) altruistic.

#### Moral Foundations Questionnaire

Moral foundations questionnaire was used to measure moral foundations (original version: Graham et al., 2011; Polish version: Jarmakowski-Kostrzanowski and Jarmakowska-Kostrzanowska, 2016; Swedish version: Nilsson and Erlandsson, 2015). The tool consists of 30 items and is a self-report measure. The participant's task is to assess the importance of five moral foundations for decision-making. The foundations are fairness/reciprocity, harm/care, authority/respect, ingroup/loyalty, and purity/sanctity. In the first part of the questionnaire (15 items), the respondents answer how relevant specific issues are in making a moral decision for them, for example, *Whether or not someone acted unfairly*. In this part, the participants answer on a six-point Likert scale ranging from 1 = not at all relevant (*This consideration has nothing to do with my judgments of right and wrong*) to 6 = extremely relevant (*This is one of the most important factors when I judge right and wrong*). In the second part (15 items), the participants agree or disagree with statements reflecting moral foundations; for example, *People should not do things that are disgusting, even if no one is harmed*. In this part, the participants answer on a six-point Likert scale ranging from 1 = *strongly disagree* to 6 = *strongly agree*. The five moral foundations are typically further classified into two groups as follows: individualizing (fairness/reciprocity, harm/care) and binding (authority/respect, ingroup/loyalty, and purity/sanctity; Van Leeuwen and Park, 2009; Garvey and Ford, 2014). As this approach is more statistically efficient and specifically serves our hypotheses testing, we embraced it. We counted general scores for Individualizing and binding moral foundations by computing the mean of relevant items for both subscales. For the Polish sample: for individualizing moral foundations, Cronbach's α = 0.81; for binding moral foundations, α = 0.85; for the Swedish sample: for individualizing moral foundations, Cronbach's α = 0.74; for binding moral foundations, α = 0.84.

#### Ecological behaviors in the last 6 months

We used a survey of our construction, which was based on one question (*In the last 6 months, to what extent have you…*) with answer options ranging from 0 = not at all to 10 = totally, asked about seven behaviors: (1) limited food waste in your household, (2) limited water waste in your household, (3) limited energy consumption in your household, (4) limited buying new clothes, (5) chosen local products over imported ones, (6) limited unnecessary travel, and (7) limited meat consumption (preceded by a question about general meat consumption and asked only to meat consumers). The global score was computed as the mean of seven items. For the Polish sample, Cronbach's α = 0.68; for the Swedish sample, α = 0.62.

#### Pro-environmental behaviors' intentions for the following 6 months

We used a survey of our construction, which was based on one question (*To what extent are you ready to undertake the following behaviors in the next 6 months*) with answer options ranging from 0 = not at all to 10 = totally, asked about seven behaviors as follows: (1) limit food waste in your household, (2) limit water waste in your household, (3) limit energy consumption in your household, (4) limit buying new clothes, (5) choose local products over imported ones, (6) limit unnecessary travel, and (7) limit meat consumption (preceded by a question about meat consumption and asked only to meat consumers). The global score was computed as the mean of seven items. For the Polish sample, Cronbach's α = 0.75; for the Swedish sample, α = 0.75.

#### Opinion about pro-environmental behavior and the pandemic threat link

We used a survey of our construction based on the following three statements: (1) *The pandemic has shown me that we should care for the environment more*, (2) *I fear that if we do not care for the environment, another pandemic may come in the future*, and (3) *I think that the COVID-19 pandemic appeared because we did not care for the environment as much as we should*. The participants answered on a 5-point Likert scale ranging from 1 = *totally disagree* to 5 = *totally agree*. The global score was computed as the mean of the three items. For the Polish sample, Cronbach's α = 0.77; for the Swedish sample, α = 0.73.

### Analytical tools

We performed all analyses using SPSS 28.0.1.0 for Windows (IBM Corp., 2021). For moderation analysis *post-hoc* tests, we also used PROCESS v4.0 for SPSS (Hayes, 2018).

## Results

### Open data note

The analysis has been preregistered (hypotheses H1–H3 and the set of covariates); the document is available from https://aspredicted.org/blind.php?x=GF6_4W3. Data underlying the project is available from Open Science Framework under the link https://osf.io/wcszy/.

### Descriptive statistics and intergroup comparisons

In Table A1, we present an overview of the descriptive statistics (mean, standard deviations, skewness, and kurtosis) regarding the variables of interest, as well as the results of the *t*-test of differences between Polish and Swedish samples in terms of these variables.

Data from Table A1 suggest that Poles and Swedes differed significantly in terms of their age, with Swedes being older, *t*_(281.56)_ = −5.82; *p* < 0.001 (degrees of freedom different than in other cases due to heterogeneous variances detected in Levene's test); present-hedonistic time perspective, with Poles having this individual difference higher than Swedes, *t*_(299)_ = 5.33; *p* < 0.001; individualizing moral foundations, with Poles having them higher than Swedes, *t*_(299)_ = 4.85; *p* < 0.001; binding moral foundations, with Poles having them higher than Swedes, *t*_(299)_ = 2.60; *p* < 0.01; and pro-environmental intentions for the next 6 months, with Poles displaying them as higher than Swedes, *t*_(299)_ = 2.09; *p* < 0.05.

Next, we performed a bivariate correlation analysis to gain insight into the associations among the investigated variables. The results of these analyses are provided in Table 1. As shown in Table 1, for the pro-environmental behaviors in the last 6 months, significant bivariate correlates were as follows: age, social value orientation, and individualizing moral foundations. Significant correlates for the pro-environmental intentions for the following 6 months were as follows: country (Poland), age, female gender, education, social value orientation, future time perspective, individualizing moral foundations, and pro-environmental behaviors in the last 6 months. For the opinion about the link between pro-environmental behaviors and the pandemic, significant correlates were female gender, social value orientation, individualizing moral foundations, pro-environmental behaviors in the last 6 months, and pro-environmental intentions for the following 6 months.

**Table 1:** Bivariate correlations and descriptive statistics between study variables.

**Variable**	**1**	**2**	**3**	**4**	**5**	**6**	**7**	**8**	**9**	**10**	**11**	**12**	**13**	**14**
1. Country (0 = Poland, 1 = Sweden)	–													
2. Age	0.32^***^	–												
3. Gender (0 = female, 1 = male)	0.15^*^	−0.06	–											
4. Education	−0.03	0.29^***^	−0.03	–										
5. Socioeconomic status	−0.07	0.02	0.05	0.18^**^	–									
6. SVO	0.05	0.05	−0.11	−0.05	0.08	–								
7. Future TP	−0.11	0.04	−0.11	0.16^**^	0.16^**^	0.07	–							
8. Present-hedonistic TP	−0.30^***^	−0.15^**^	−0.03	−0.09	−0.03	−0.03	−0.23^***^	–						
9. Present-fatalistic TP	−0.07	−0.08	−0.02	−0.16^**^	−0.15^**^	−0.01	−0.36^***^	0.47^***^	–					
10. MFQ—individualizing	−0.27^***^	0.04	−0.31^***^	0.07	−0.04	0.31^***^	0.13^*^	0.17^**^	0.00	–				
11. MFQ—binding	−0.15^**^	−0.03	0.23^***^	−0.01	0.02	−0.09	0.19^***^	0.17^**^	0.06	0.03	–			
12. Pro-environmental behaviors—past 6 months	0.03	0.15^**^	−0.10	0.06	−0.05	0.15^**^	0.10	0.02	−0.06	0.18^**^	−0.07	–		
13. Pro-environmental intentions—next 6 months	−0.12^*^	0.14^*^	−0.20^***^	0.12^*^	−0.01	0.20^***^	0.16^**^	0.06	−0.08	0.31^***^	−0.08	0.74^***^	–	
14. Pandemic threat-pro-environmental behaviors link opinion	0.00	−0.03	−0.25^***^	−0.10	−0.02	0.12^*^	0.01	0.07	−0.01	0.25^***^	−0.06	0.26^***^	0.25^***^	–

*** p < 0.001.

** p < 0.01.

* p < 0.05.

Next, to perform the preregistered analysis, we ran three moderation models with bootstrapping (*N* = 5,000) for the following dependent variables: pro-environmental behaviors score for the last 6 months (Model 1), pro-environmental intentions score for the following 6 months (Model 2), and the opinion about the threat of pandemic related to environmental issues (Model 3). The main aim of the preregistered moderation analysis was to test the significance of the social value orientation × future time perspective × country interaction. Given the results of the correlation analysis, only the preregistered covariates, which were significant correlates in our sample, were entered into the models. All variables were standardized before entering them into the models; interactions were computed on centered variables. The results of moderation analyses are presented in Tables 2–4.

**Table 2:** Results of moderation analysis predicting pro-environmental behaviors in the last 6 months.

**Predictors**	***B* (95% CI)**	**SE**	***t***	***p*-value**
Future time perspective	0.07 (−0.04; 0.19)	0.06	1.17	0.242
Social value orientation	0.09 (−0.03; 0.21)	0.06	1.47	0.144
Country (0 = Poland, 1 = Sweden)	0.03 (−0.09; 0.14)	0.06	0.40	0.687
Future time perspective × Social value orientation	−0.10 (−0.24; −0.01)	0.05	−1.97	0.050
Future time perspective × Country	−0.05 (−0.16; 0.06)	0.06	−0.95	0.342
Social value orientation × Country	0.06 (−0.04; 0.18)	0.06	1.13	0.262
Social value orientation × Future time perspective × Country	0.07 (−0.07; 0.17)	0.05	1.33	0.184
Age	0.14 (0.03; 0.25)	0.06	2.29	0.022
Individualizing moral foundations	0.14 (0.02; 0.26)	0.06	2.30	0.022
$R_{\text{adj}}^{2}$	0.064			
*F*_(9;291)_	3.27			
*p*-value	< 0.001			

**Table 3:** Results of moderation analysis predicting pro-environmental intentions for the next 6 months.

**Predictors**	***B* (95% CI)**	**SE**	***t***	***p*-value**
Future time perspective	0.08 (−0.02; 0.22)	0.06	1.45	0.148
Social value orientation	0.12 (0.01; 0.24)	0.06	1.93	0.054
Country (0 = Poland, 1 = Sweden)	−0.10 (−0.22; 0.02)	0.06	−1.59	0.113
Future time perspective × Social value orientation	−0.02 (−0.17; 0.08)	0.05	−0.40	0.691
Future time perspective × Country	−0.03 (−0.14; 0.09)	0.06	−0.57	0.571
Social value orientation × Country	0.03 (−0.09; 0.14)	0.06	0.45	0.653
Future time perspective × Social value orientation × Country	0.00 (−0.13; 0.11)	0.05	0.00	0.999
Age	0.13 (.02; 0.25)	0.06	2.17	0.031
Gender (0 = female, 1 = male)	−0.09 (−0.20; 0.02)	0.06	−1.58	0.115
Education	0.05 (−0.09; 0.20)	0.06	0.93	0.353
Individualizing moral foundations	0.19 (0.07; 0.31)	0.06	3.06	0.002
$R_{\text{adj}}^{2}$	0.120			
*F*_(11;285)_	4.65			
*p*-value	< 0.001			

**Table 4:** Results of moderation analysis predicting the opinion about the pandemic threat and pro-environmental behaviors' link.

**Predictors**	***B* (95% CI)**	**SE**	***t***	***p*-value**
Future time perspective	−0.04 (−0.17; 0.10)	0.06	−0.77	0.442
Social value orientation	0.02 (−0.10; 0.13)	0.06	0.36	0.721
Country (0 = Poland, 1 = Sweden)	0.08 (−0.04; 0.20)	0.06	1.33	0.185
Future time perspective × Social value orientation	−0.03 (−0.16; 0.08)	0.05	−0.54	0.593
Future time perspective × Country	0.05 (−0.08; 0.18)	0.06	0.83	0.410
Social value orientation × Country	−0.04 (−0.15; 0.07)	0.06	−0.71	0.481
Future time perspective × Social value orientation × Country	−0.06 (−0.20; 0.04)	0.05	−1.09	0.278
Gender (0 = female, 1 = male)	−0.20 (−0.32; −0.09)	0.06	−3.39	< 0.001
Individualizing moral foundations	0.21 (0.08; 0.32)	0.06	3.27	0.001
$R_{\text{adj}}^{2}$	0.085			
*F*_(9;287)_	4.05			
*p*-value	< 0.001			

According to Table 2, the overall regression was statistically significant, *F*_(9;291)_ = 3.27, *p* < 0.001, $R_{\text{adj}}^{2}$ = 0.064. The statistically significant predictors of pro-environmental behaviors in the last 6 months were as follows: the interaction between future time perspective and social value orientation, *B* = −0.10; 95% CI (−0.20; −0.00), age, *B* = 0.14; 95% CI (0.02; 0.26) and individualizing moral foundations, *B* = 0.14; 95% CI (0.02; 0.27). Due to one significant interaction found in the analysis, we performed *a post-hoc* analysis. They indicated that the link between future time perspective and pro-environmental behaviors in the last 6 months was statistically significant only for low social value orientation, *B* = 0.17, 95% CI (0.02; 0.32), whereas it was insignificant for average, *B* = 0.07; 95% CI (−0.05; 0.18) and high, *B* = −0.03; 95% CI (−0.19; 0.12) social value orientation.

As indicated in Table 3, the overall regression was statistically significant, *F*_(11;285)_ = 4.65, *p* < 0.001, $R_{\text{adj}}^{2}$ = 0.120. The statistically significant predictors of pro-environmental intentions for the following 6 months were as follows: age, *B* = 0.13; 95% CI (0.02; 0.25) and individualizing moral foundations, *B* = 0.19, 95% CI (0.07; 0.32).

Data in Table 4 suggest that the overall regression was statistically significant, *F*_(9;287)_ = 4.05, *p* < 0.001, $R_{\text{adj}}^{2}$ = 0.085. The statistically significant predictors of the opinion about the link between the pandemic and pro-environmental behavior were as follows: female gender, *B* = −0.20; 95% CI (−0.32; −0.09) and individualizing moral foundations, *B* = 0.21, 95% CI (0.08; 0.32).

## Discussion

In the current study, we aimed to investigate the role of future time perspective, social value orientation, and their interaction in predicting the following: (1) pro-environmental behaviors in the last 6 months, (2) pro-environmental behavior intentions in the following 6 months, and (3) opinion about the linkage between pro-environmental attitude and the threat of pandemics. We also intended to control for individualizing/binding moral foundations, present time perspectives, and demographic variables, including age, gender, education, and socioeconomic status, to determine whether the hypothesized interaction was significant above these variables.

As judged by the correlation analyses, the data supported H1 about the positive relationship between social value orientation and the outcome variables. A significant positive association was observed for pro-environmental behaviors, intentions, and opinions, which is consistent with prior research (De Groot and Steg, 2010), even though in none of the cases, this simple effect remained significant when controlling for other variables of interest. Similarly, the bivariate analyses revealed that future time perspective was positively associated with pro-environmental intentions, which aligns with prior research (Milfont et al., 2012). However, this simple effect was no longer observed in the multivariate analyses. Interactions hypothesized in H2 and H3 could explain the lack of simple effects.

H2 and H3 referred to the potential differences between Poland and Sweden regarding the future time perspective and social value orientation interaction in predicting pro-environmental behaviors/intentions/opinions. Our results were contrary to both hypotheses. The participants' country of origin did not play a moderating role in the models. As social value orientation and future time perspective are individual differences, present regardless of the culture, their interaction mechanisms may translate into similar outcomes. However, given that Poland and Sweden are part of the W.E.I.R.D. world (Henrich et al., 2010), further studies are needed to investigate whether the effects pertain to non-Western countries.

The lack of difference between Poland and Sweden may also stem from the lack of difference between our samples in terms of the levels of future time perspective, social value orientation, pro-environmental behaviors in the last 6 months, and the opinions about the link between pro-environmental behaviors and the pandemic threat. The specificity of online panel users might partially explain it. Such panels attract active users of the Internet, who, at the same time, wish to earn some small sums of money for their survey participation. It may be the reason for the similarity of samples in terms of the investigated mechanisms. However, the study revealed some interesting differences between our samples. First, Poles were younger and more Present-Hedonistic. These two characteristics are typically associated (Laureiro-Martinez et al., 2017), as younger people are more risk-taking and pleasure-oriented than older people.

Moreover, Poles were higher on both individualizing and binding morality. Poland might have a higher social desirability bias regarding morality-related statements, as it is a less secularized country than Sweden (Demerath, 2000). Detachment from religion in Sweden might encourage people to self-report their moral convictions more carefully. Moreover, as Poland is a more conservative society at large than Sweden, the integration between individualizing and binding morality might be higher in Polish society (Turner-Zwinkels et al., 2021).

Finally, Poles had greater pro-environmental intentions for the following 6 months than Swedes, which is in line with a recent study by Iwińska et al. (2023) about the pro-environmental behaviors during COVID-19 in Europe. It might be related to the economic concerns related to inflation, as in Poland, the harmonized inflation rate for 2022 was 13.15%. In contrast, in Sweden, it was 8.04% (Worldwide Inflation Data, 2023). Poles, therefore, might have thought more about ways to reduce their expenses in the nearest future, and the behaviors we asked about were one of the ways to do so.

A two-way future time perspective and social value orientation interaction were statistically significant for the past pro-environmental behaviors in the last 6 months' model. It was not observed for other models. The *post-hoc* tests indicated that the future time perspective activates only when social value orientation is low. Thus, the norm activation model (Schwartz, 1970) notions were not fully confirmed. In our study, people low on social value orientation are focused on the benefits to self (Murphy and Ackermann, 2014). For these people, future time perspective can activate pro-environmental behaviors due to thinking about the positive consequences, for example, saving money (Rolison et al., 2017). It is because pro-environmental behaviors may be motivated by a desire to save money by cutting down expenses (e.g., by saving energy or water or avoiding meat consumption). Notably, 6 months before the study referred to a period of post-COVID and then the war in Ukraine-related inflation in Europe, which strongly encouraged people to save money and energy. It could translate into the effect observed in our study.

Consistent with H4, individualizing moral foundations positively predicted pro-environmental behaviors, intentions, and opinions; moreover, they seemed to be the strongest predictor of all investigated. It aligns with previous data (Vainio and Mäkiniemi, 2016; Milfont et al., 2019). However, contrary to the second part of H4, binding moral foundations were not significantly linked to any of the examined dependent variables. It suggests that rather than cooperation or ingroup, loyalty or sanctity valuing tendencies, the moral imperative of caring for other individuals can be universal in predicting environmental care and feeling threatened by pandemics. It is in line with previous research on environmental concerns and moral foundations by Milfont et al. (2019). It shows an interesting avenue for future research and formulating messages highlighting individual testimonies or individual-level consequences when promoting environmental actions.

Regarding the demographic variables, the female gender was significantly associated with pro-environmental intentions for the next 6 months, and opinions about the pandemic threat and pro-environmental behaviors link. For the latter outcome variable, the gender association remained significant in models including the entire set of predictors. These findings are consistent with the evidence of stronger environmental attitudes and behaviors in females reported elsewhere (e.g., Zelezny et al., 2000). Research shows that women feel more threatened by the COVID-19 pandemic (Luo et al., 2021). Also, in our study, older age was a unique predictor of pro-environmental behaviors and intentions. This pattern aligns with meta-analytic evidence that older adults are slightly more likely than younger adults to engage in nature, avoid environmental harm, and conserve raw materials (Wiernik et al., 2013). However, no significant effect was observed in bivariate analyses for sociodemographic status and no effect in multivariate analyses for education. The reason behind this could be the recruitment strategy—Prolific.co panelists may be specific regarding their sociodemographic characteristics, and they do not constitute a representative sample. However, given that pro-environmental behaviors are, on the one hand, beneficial to the family budget (saving) and, on the other—money consuming (investment in eco-friendly products), the motivation behind them might be different in people of different socioeconomic status/education. Therefore, the simple effects of these demographic variables disappear.

## Limitations of the study and future research directions

Although our study provides some interesting insight into pro-environmental behaviors and intentions, it has limitations that should be considered. First, the study was performed online with limited control over how attentive the respondents answered the survey. It was also purely questionnaire- and declaration-based, potentially producing self-report or social desirability bias. Observational, experimental, intervention, or multi-method studies would be a way to corroborate further the results and conclusions drawn.

Moreover, the participants were recruited with a method of convenience sampling and only registered users of Prolific.co, which formed a specific and non-representative study sample. Despite targeting a broad audience, this data collection method limited the chances of capturing the full complexity of human behavior. Furthermore, this study involves cross-sectional analyses, precluding firm conclusions regarding the causal mechanisms involved. To overcome this limitation, future studies should employ longitudinal designs to, for example, examine the cross-lagged associations of variables. An exciting avenue would be ecological momentary assessment or diary studies on pro-environmental behaviors, which could help us determine how people behave in specific timeframes.

Furthermore, our study relied on a selected theoretical framework, broadened based on the literature review and the new avenues emerging in the field. Given the promising role of individualizing moral foundations in predicting pro-environmental behaviors/intentions/opinions, future studies should continue to examine this variable to deepen the understanding of the mechanisms underlying this link. Other relevant theoretical frameworks could be applied to enhance the robustness of the analyses.

We gathered data from two countries differing in the quality of their green policies and the level of support for pro-environmental causes in current politics. Future studies could control for more specific factors helpful in determining under what conditions future time perspective shapes pro-environmental opinions/behaviors. From the contextual level, it could be the general income in a country, wealth of a place of living, inflation indices at the time of conducting the study, indicators of green policies in the place of residence, and measures of state support for the environment. Furthermore, from the individual level, the propensity to save money or to spend it on valued causes could be used as potential covariates/moderators of the future time perspective effect on pro-environmental opinions/behaviors.

## Implications for practice

Our data supported the idea regarding the role of future time perspective for green behaviors only in the case of past pro-environmental behaviors. Regardless of the participant's country of origin, future time perspective was related to pro-environmental behaviors in the last 6 months only when social value orientation was low. It suggests that not the cultural aspects but the level of orientation toward others' welfare plays a role in the case of this behavior. For example, the results might be used when advertising pro-environmental behaviors and designing campaigns. When encouraging more competitive (compared to altruistic) people to behave in a green way, it might be crucial to underline the future consequences and benefits, consistent with the future time perspective. The pro-environmental campaigns could, therefore, highlight how green behavior may bring personal gains in the future, which are typically valued by individualistic people, such as savings or social status. Existent pro-environmental programs based on competitiveness, such as Greencoin (Duda et al., 2022), which, based on the mobile application, encourages learning and reporting own green behaviors to obtain rewards, can be a good example of a way to go in order to encourage competitive people to behave pro-environmentally. Moreover, individualizing moral foundations of care and justice concerns appeared to predict behaviors, intentions, and opinions regarding pro-environmental issues. When educating and raising pro-environmental awareness, activation of this kind of morality may help promote green behaviors. This could happen by highlighting how attentiveness to the environment can contribute to caring for vulnerable members of society or how fair it is in the context of the community. For example, it is worth showing that behaving pro-environmentally encourages social equality and supports individual people's welfare.

## Author's note

The study has been preregistered with AsPredicted.org: https://aspredicted.org/blind.php?x=GF6_4W3.

## Data availability statement

The datasets presented in this study can be found in online repositories. The names of the repository/repositories and accession number(s) can be found at: https://osf.io/wcszy/ (Open Science Framework).

## Ethics statement

The studies involving humans were approved by Research Ethics Committee at The Maria Grzegorzewska University, approval number 89/2022. The studies were conducted in accordance with the local legislation and institutional requirements. The participants provided their written informed consent to participate in this study.

## Author contributions

IN: conceptualization, methodology, validation, formal analysis, investigation, resources, data curation, writing—original draft, writing—review and editing, visualization, supervision, project administration, and funding acquisition. MR: conceptualization, methodology, writing—original draft, and writing—review and editing. All authors contributed to the article and approved the submitted version.

## Appendix

**Table A1:** Descriptive statistics of variables of interest and intergroup comparisons.

**Variable**	**Polish sample**				**Swedish sample**				***t***	***p***
	***M***	***SD***	**Skewness**	**Kurtosis**	***M***	***SD***	**Skewness**	**Kurtosis**		
Age	25.45	7.55	1.79	3.19	31.32	9.81	1.25	1.17	–5.82	< 0.001
Education	14.99	2.13	0.51	–0.41	14.84	2.99	0.24	1.88	0.49	0.626
Socioeconomic status	5.81	1.74	–0.34	0.08	5.52	2.36	–0.11	–0.45	1.21	0.226
SVO	30.91	12.27	–0.98	0.76	32.22	13.00	–1.50	3.11	–0.90	0.370
Future TP	3.40	0.62	–0.59	0.21	3.27	0.55	0.03	0.07	1.95	0.052
Present-hedonistic TP	3.32	0.53	0.19	–0.07	2.96	0.60	–0.04	–0.21	5.44	< 0.001
Present-fatalistic TP	2.77	0.61	0.26	–0.10	2.69	0.63	0.09	–0.45	1.18	0.240
MFQ—individualizing	4.77	0.63	–0.81	1.40	4.42	0.64	–0.16	–0.11	4.85	< 0.001
MFQ-binding	3.26	0.70	0.04	–0.21	3.04	0.70	0.04	–0.33	2.69	0.008
Pro-environmental behaviors—past 6 months	5.51	1.67	–0.70	0.61	5.63	2.00	–0.26	–0.67	–0.60	0.551
Pro-environmental intentions—next 6 months	6.39	1.79	–0.89	1.22	5.95	1.86	–0.66	0.06	2.09	0.037
Pandemic threat-pro-environmental behaviors link opinion	2.76	1.04	–0.19	–0.64	2.76	1.15	0.27	–0.79	–0.03	0.976
